# The Development of the Guide to Economic Analysis and Research (GEAR) Online Resource for Low- and Middle-Income Countries’ Health Economics Practitioners: A Commentary

**DOI:** 10.1016/j.jval.2017.10.003

**Published:** 2018-05

**Authors:** Chiaki Urai Adeagbo, Waranya Rattanavipapong, Lorna Guinness, Yot Teerawattananon

**Affiliations:** Health Intervention and Technology Assessment Program, Department of Health, Ministry of Public Health, Nonthaburi, Thailand

**Keywords:** cost, cost-effectiveness analysis, economic evaluation, health technology assessment, low- and middle-income countries

## Abstract

Public health authorities around the world are increasingly using economic evaluation to set priorities and inform decision making in health policy, especially in the development of health benefit packages. Nevertheless, researchers in low- and middle-income countries (LMICs) encounter many barriers when conducting economic evaluations. In 2015, the Health Intervention and Technology Assessment Program identified key technical and context-specific challenges faced in conducting and using health economic evaluations in LMICs. On the basis of these research findings, the Guide to Economic Analysis and Research (GEAR) online resource (www.gear4health.com) was developed as a reliable aid to researchers in LMICs that would help overcome those challenges. Funded by the Thailand Research Fund and the Bill and Melinda Gates Foundation, GEAR is a free online resource that provides a visual aid tool for planning economic evaluation studies (GEAR mind maps), a repository of national and international economic evaluation guidelines (GEAR guideline comparison), and an active link to a network of volunteer international experts (GEAR: Ask an expert). GEAR will evolve over time to provide relevant, reliable, and up-to-date information through inputs from its users (e.g., periodic survey on methodological challenges) and experts (e.g., in responding to users’ questions). The objective of this commentary was to give a brief description of the development and key features of this unique collective information hub aimed at facilitating high-quality research and empowering health care decision makers and stakeholders to use economic evaluation evidence.

## Introduction

A critical component in health care priority setting, in particular in the development of benefit packages, is the assessment of the value for money. For this reason, health care decision makers use economic evaluation as a tool for comparing the costs and benefits of health care interventions. Unlike health systems in many high-income countries (HICs) with formal health technology assessment (HTA) frameworks that incorporate economic evaluations, these frameworks are not well formalized in low- and middle-income countries (LMICs). LMICs face a number of limitations in conducting economic evaluation as well as in applying economic evaluation study results in policymaking. A key challenge is the difficulty in obtaining data and the limited capacity to conduct economic evaluation studies [1], [2], [3]. Studies conducted in LMICs note that there is a lack of trained researchers with analytic skills and experience [4], [5], [6], [7], [8], [9], unavailability of methodological guidelines [4], [5], absence of an institutionalized research environment [4], [5], [6], [7], [8], [9], and less awareness of application of evidence-based policymaking among researchers and decision makers [5], [6], [7], [8], [9]. Furthermore, incorporating economic evaluation results into resource allocation processes is constrained by both a lack of decision makers’ understanding of economic evaluation and the social, political, and ethical factors that influence these decisions [10].

International initiatives sch as the Disease Control Priorities project [11] and World Health Organization’s (WHO’s) initiative WHO-CHOICE (CHOosing Interventions that are Cost-Effective) [12] have attempted to overcome some of these challenges. Both have developed methods and provided data on the cost-effectiveness of various health care interventions that treat or prevent diseases resulting in the major health burdens in broad geographical regions. Nevertheless, these attempts have focused on the technical aspects of defining globally recommended cost-effective health care interventions and less on building local capacity around processes—from collecting data to applying the results of analysis in policymaking [13]. The International Decision Support Initiative (iDSI), a global partnership that supports health care policy decision making, has now published a reference case that outlines a set of key principles for the conduct and reporting of economic evaluation studies [14]. This reference case aims to enhance comparability across studies while allowing for flexibility of individual, institutional, or political value judgments to facilitate domestic decision making in LMICs [15]. None of these initiatives address the issue of how to design and conduct economic evaluations in resource-constrained settings in which there can be issues around data availability and access as well as political will and know-how [3]. To complement these initiatives, the Guide to Economic Analysis and Research (GEAR)—an online resource developed by the Health Intervention and Technology Assessment Program (HITAP) (www.gear4health.com)—intends to become an important aid to HTA practitioners in LMICs. Available free of charge, GEAR provides a guide to addressing the challenges that researchers in LMICs face when conducting and using economic evaluations. The objective of this commentary was to give a brief description on the development and key features of the GEAR online resource.

## Developing GEAR—Consulting with Practitioners and Users

In 2015, HITAP conducted research to identify the most important difficulties and barriers faced by researchers in LMICs when conducting economic evaluations for policymaking in health care [16]. The work included a literature search and a subsequent survey among 110 qualified respondents from 35 different countries who had completed at least one economic evaluation project in an LMIC setting. Luz et al. [16] categorized the difficulties identified from the literature search into technical or context-specific difficulties for inclusion in the survey. Technical difficulties were defined as those that directly related to the methodology used in an analysis and could be overcome by learning new tools and techniques. Context-specific difficulties were issues that indirectly affected the studies and how they were used, varying by country or setting, and often outside the control of the study. The results from the survey showed that the top five technical difficulties were 1) lack of high-quality local clinical data, 2) poor reporting on economic evaluations, 3) lack of data on the costs from various perspectives (patient, societal, government, etc.), 4) paucity of commonly accepted methodological guidelines, and 5) lack of local data for estimating quality-adjusted life-years (QALYs) and disability-adjusted life-years (DALYs). The most important contextual issues were 1) the noninclusion of economic evaluations in the decision-making process, 2) limited local research capacity, 3) lack of funding, 4) weak communication between researchers and end users of the evidence, and 5) limited number of published local journals. Sixty-six percent of respondents considered that context-specific issues, such as the exclusion of economic evaluation from the decision-making process, lack of funding for the research, and misunderstandings and weaknesses in the communication between researchers and relevant stakeholders, were more impeding than technical issues.

As a result of this work, HITAP held a consultation with world-leading research partners and policymakers from universities, ministries of health, HTA agencies, and the WHO to discuss the findings and ways to address the challenges that were found. The GEAR online resource was born. The tool has been devised to tackle both technical and contextual difficulties focusing on the short-term but with the long-term in mind. For each of the technical and context-specific issues, short-term advice is provided in the form of currently available solutions to the problems on the basis of the latest evidence and examples of best practice. For the longer term, GEAR identifies and encourages further research in key areas in which it is known that there is insufficient empirical research. GEAR will use three key features to support HTA practitioners in overcoming the challenges that they face in doing better quality research: GEAR mind maps, GEAR guideline comparison, and GEAR: Ask an expert. Each of these tools will evolve over time to address user recommendations and incorporate frequently asked questions into its knowledge base.

### GEAR Mind Maps: A Visual Tool for HTA Practitioners

The GEAR mind maps are designed to enable users to visualize the process involved in economic evaluation by presenting ideas in a tree-like structure. The core topic of each mind map is a technical or contextual issue identified in the previous research. The topic is placed at the center of the mind map, with branches and sub-branches extending out from the center to describe associated issues. There are two main branches: solutions (right-hand side) and unanswered research questions (left-hand side). The right-hand side of each mind map presents solutions to the selected problem, where known solutions exist. The map signposts users through decisions that need to be made in a study design, presenting the alternative solutions available and guidance on when each solution is appropriate. In addition, the map provides references to the supporting literature such as textbooks and published articles (short-term solutions). The framework for the right-hand side is based on the collective knowledge of experts in research and teaching economic evaluation in LMICs. The left-hand side of each mind map identifies areas within a topic for which more evidence and research are needed and presents research questions that could address these difficulties (long-term solutions). These research questions are designed to shape methodological judgments in the long-term and could be addressed by the GEAR audience in their work or included in future GEAR surveys.

**Fig. 1:**
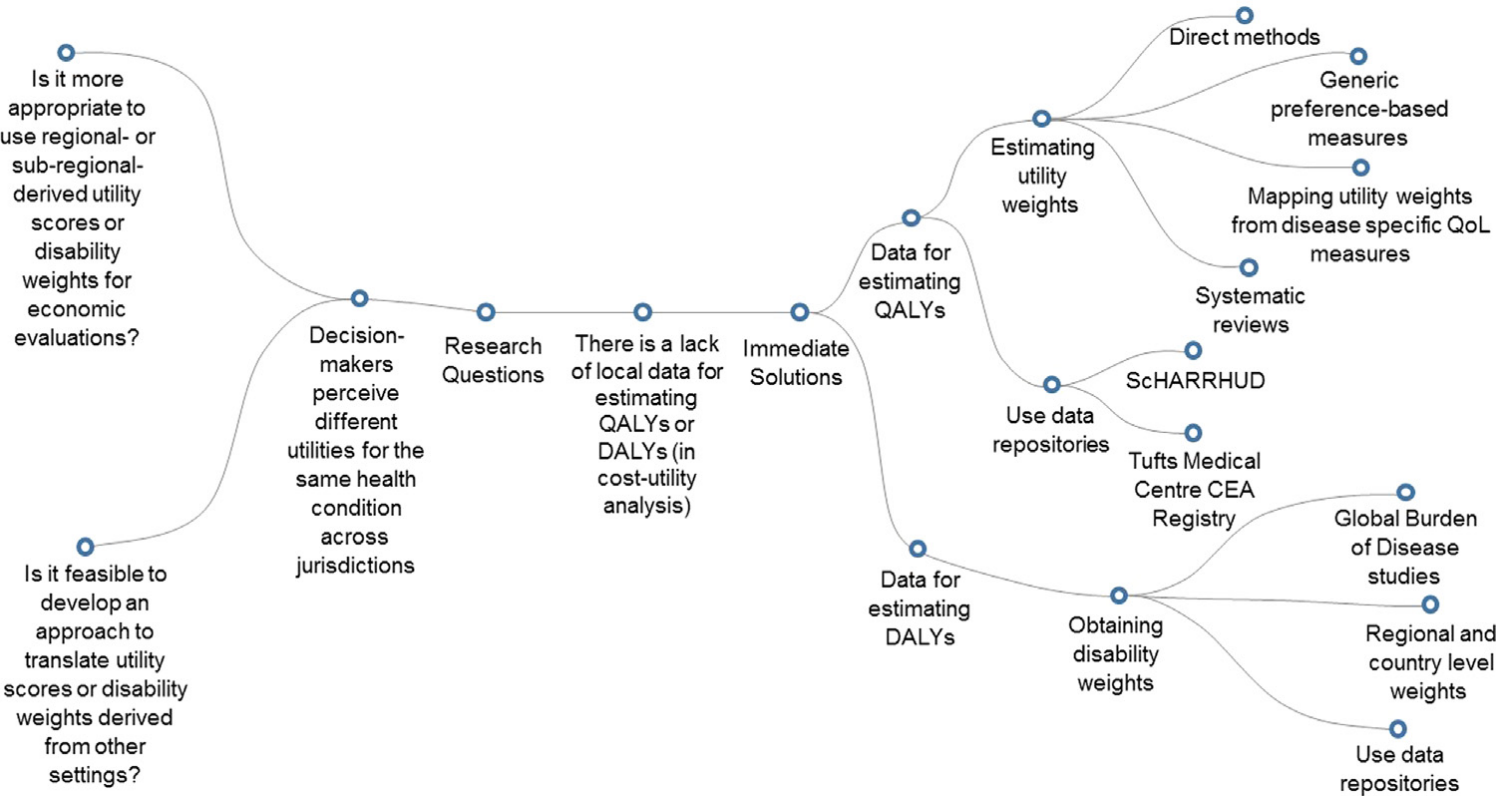
Mind map addressing a lack of local data for estimating QALYs or DALYs (in cost-utility analysis). The right side of the mind map provides immediate solutions, whereas the left side presents unanswered research questions when methodological uncertainty still exists. CEA, cost-effectiveness analysis; DALY, disability-adjusted life-year; QALY, quality-adjusted life-year; QOL, quality of life; ScHARRHUD, School of Health and Related Research Health Utilities Database.

In the first phase, the GEAR mind maps have been developed to address the top five technical difficulties derived from the original HITAP survey. Each of the mind maps will evolve with new nodes and links being added as methodological fields also move forward. For example, if users need utility data for their calculation of QALYs and are unsure about where to obtain these data from, they would choose the mind map “Addressing a lack of utility data to calculate QALYs or DALYs,” as shown in Figure 1. In this example, the “solutions” side (right-hand side) enables users to explore available tools and approaches, such as an introduction to generic preference-based measures and links to where you can find these measures or more information about them. Nevertheless, the researchers may feel that it is not appropriate to use global utility weights in their analysis. In exploring the mind map they will see that the question “Is it more appropriate to use regional- or subregional-derived utility scores or disability weights for economic evaluations?” is on the left-hand side, noting an area where knowledge is scarce and that there is a need for a study in this local area.

### GEAR Guidelines Comparison: A Repository for Economic Evaluation Guidelines

The GEAR guideline comparison section hosts a repository of national and international economic evaluation guidelines that will be updated periodically (approximately every 6 months). Guidelines are identified using an online search of Google Scholar and MEDLINE (Medical Subject Headings keywords: Guideline AND Cost-Benefit Analysis). On the GEAR Web site, the guidelines are classified according to their country’s economic income level, in line with 2016 World Bank classification [17]. When LMICs do not have country-specific guidelines, they may want to borrow from countries with similar settings or need to reference LMIC-specific guidelines. In selecting guidelines, it is important to understand the economic evaluation guidelines in terms of their methodological specification and perspectives, and whether they are for use at the country, regional, or international level. This is because many existing guidelines are developed by and for HIC settings with specific health system designs and purchasing procedures that shape the methodology of an economic evaluation. Guidelines from HIC settings therefore maybe less applicable in an LMIC.

The guideline comparison tool enables users to compare selected guidelines in terms of specific topics (e.g., health outcome measure) using a “query box” to explore the acceptability of these guidelines. The guidelines are compared across topics of critical interest in designing and carrying out economic evaluations. In the first phase of GEAR, the comparison tool includes the guidelines produced by international organizations such as the WHO and the iDSI, because both are designed for use in LMICs [15], [18]. To reflect the comparative perspective from country settings, national guidelines were selected from two countries: the United Kingdom (where the National Institute for Health and Care Excellence is a global leader in economic evaluation for health care decision making) [19] and Thailand (the first LMIC to develop its own guidelines) [20]. More countries will be added to the comparison tool as GEAR is updated.

Finally, this section also hosts the International Society for Pharmacoeconomics and Outcomes Research series of reports “ISPOR Good Practices for Outcome Research” [21], which provide consensus around key methodological issues in economic evaluation.

### Interactive GEAR: Ask an Expert

“Ask an expert,” the third feature of GEAR, allows registered users to ask advice from an international expert. GEAR will maintain a network of volunteer experts who agree to answer questions from the users. Users may post a question on the GEAR platform related to conducting economic evaluations. The GEAR manager will screen the question and send it to two or three volunteer experts who specialize in the field related to the question. The experts are requested to provide an answer within a limited time frame, of which the user will be notified. Experts will specialize in a wide range of fields, covering clinical outcomes, costing, decision analytic modeling, health policy, and utility measures. The answers from experts will be posted on to the platform and a discussion thread started. Subsequently, all registered users will be able to add their ideas, opinions, and experience to this thread. In this way, GEAR encourages learning, sharing, and debate. The questions and answers will be archived and made available for searching through keywords.

## Monitoring and Adapting GEAR: In-Built Evaluation

GEAR monitors and collects information from user activity (number of visits, channel of access, devices used to access the Web site, geographical information of the users, and basic characteristics of GEAR subscribers, who want to post questions or receive information about future updates) so that it can better reflect the needs of researchers in LMICs. There will be a comprehensive evaluation of GEAR in the near future to learn about its applicability and impact. Bi-annual surveys of users will aid in further understanding of the current challenges in these regions. In addition, external experts from universities and other HTA institutions worldwide will assess the technical contents on the Web site to authenticate the credibility of the information provided.

## GEAR: Future Implications

The goal of GEAR is to become a unique collective information hub for health economist practitioners such as researchers, academics, and ministry of health employees in LMICs, helping to fill a gap in capacity in these settings. The information on the Web site will be expanded and updated regularly to provide reliable and up-to-date information to users as methodological standards develop or new methodological barriers are identified. For example, new mind maps will be developed to address contextual problems and other issues identified by GEAR users. To address the problems of Internet access in LMICs, iDSI is considering solutions such as providing downloadable mind maps and working with the developer to minimize bandwidth requirements for viewing the Web site. To increase uptake and ensure the relevance of the site to its intended users, GEAR has developed collaborations with international and regional professional associations including HTAsiaLink (www.htasialink.org) and global initiatives such as the Global Health Cost Consortium (www.ghcosting.org), and it is continuing to establish further partnerships. Through making good quality support in economic evaluation available to a global audience, we hope that the GEAR Web site will foster high-quality research and facilitate better decision making in health care in LMICs.
